# Live vaccines against bacterial fish diseases: A review

**DOI:** 10.14202/vetworld.2019.1806-1815

**Published:** 2019-11-21

**Authors:** Aslizah Mohd-Aris, Mohd Hafiz Ngoo Muhamad-Sofie, Mohd Zamri-Saad, Hassan Mohd Daud, Md. Yasin Ina-Salwany

**Affiliations:** 1 Department of Biology, School of Biology, Faculty of Applied Sciences, Universiti Teknologi MARA, Cawangan Negeri Sembilan, Kampus Kuala Pilah, Malaysia; 2 Laboratory of Marine Biotechnology, Institute of Bioscience, Universiti Putra Malaysia, Serdang, Malaysia; 3 Department of Veterinary Pathology and Microbiology, Faculty of Veterinary Medicine, Universiti Putra Malaysia, Serdang, Malaysia; 4 Department of Veterinary Clinical Studies, Faculty of Veterinary Medicine, Universiti Putra Malaysia, Serdang, Malaysia; 5 Department of Aquaculture, Faculty of Agriculture, Universiti Putra Malaysia, Serdang, Malaysia

**Keywords:** aquaculture, attenuated vaccines, bacterial fish diseases, vaccination

## Abstract

Fish diseases are often caused either by bacteria, viruses, fungi, parasites, or a combination of these pathogens. Of these, bacterial fish diseases are considered to be a major problem in the aquaculture industry. Hence, the prevention of such diseases by proper vaccination is one of the integral strategies in fish health management, aimed at reducing the fish mortality rate in the aquaculture farms. Vaccination offers an effective yet low-cost solution to combat the risk of disease in fish farming. An appropriate vaccination regime to prevent bacterial diseases offers a solution against the harmful effects of antibiotic applications. This review discusses the role of live-attenuated vaccine in controlling bacterial diseases and the development of such vaccines and their vaccination strategy. The current achievements and potential applications of live-attenuated and combined vaccines are also highlighted. Vaccine development is concluded to be a demanding process, as it must satisfy the requirements of the aquaculture industry.

## Introduction

Aquaculture contributes significantly to the global production sector, particularly in meeting the increased demand for high-quality food. Approximately 44% of the total global fish production is contributed by aquaculture [1]. As reported by Food and Agriculture Organization [2], the majority of the fish produced by aquaculture is used for human consumption. Therefore, to meet the market demands, several difficulties need to be addressed by the aquaculture sector, including natural variables such as ecological impacts, poor water quality, and disease infestations [3-5]. The current strategy to increase aquaculture productivity is based on intensification and increased commercialization of aquaculture products [6]. However, efforts for rapid intensification by aquaculture sectors may have adverse ramifications, such as disease outbreaks [7], which are a major impediment to the growth of aquaculture [8].

Disease outbreaks have socio-economic impacts since the cultures of many aquatic species sustain severe losses. There might be a loss of investment and consumer confidence, food shortage due to industry failure, or cessation of aquaculture operations [6]. Consequently, the production rate, income ability, power of employment, market access or market shares can be affected. Several cases of disease outbreaks, particularly in the Asia-Pacific region, have been reported. For instance, more than 30% of the total yield loss was estimated in China, India, and Vietnam, due to fish diseases [9]. In the Philippines, fish diseases have resulted in a 75% reduction in household income, and a 19.4% increase in debt [10]. Moreover, it has been reported the rainbow trout (*Oncorhynchus mykiss*) industry losing its sale for 29.1 million fish during 2018, which 92% loss incurred, due to diseases [11].

Many factors contribute to the susceptibility of cultured fish to pathogens. In particular, the viability of pathogens inside the fish and in the water sources often increases the chances of infection [6]. A study by Albert and Ransangan [5] revealed that deterioration of water quality can increase the susceptibility of fish to infection and diseases such as vibriosis. They reported an increase in fish mortality during periods of high water temperature, which was consistent with high counts of *Vibrio* bacteria in the diseased fish, water column, and biofilm [5]. Furthermore, Bowater *et al*. [4] suggested that pollutants, such as heavy metals, present in the environment may increase the hosts’ susceptibility to disease. According to Shefat [12], bacterial diseases are most prevalent in farmed fish. Previous reports on bacterial fish diseases have suggested motile aeromonad septicemia, edwardsiellosis, flexibacteriosis, columnaris, yersiniosis, and bacterial gill disease as the most common diseases [13]. The bacterial strains capable of predisposing the host to disease are referred to as primary pathogens, which are not always host-specific. Some bacterial strains, particularly those infecting already weakened or damaged hosts, are categorized as opportunistic pathogens [14].

In cases of vibriosis in Malaysia, *Vibrio harveyi* was most frequently isolated, followed by *Vibrio parahaemolyticus*, *Vibrio alginolyticus*, and *Vibrio anguillarum* [5]. *Vibrio* species, including *V. alginolyticus*, *V. harveyi*, and *V. parahaemolyticus*, were also found to be the causative agents infecting large yellow croakers (*Pseudosciaena crocea* (Richardson)) in China [15]. Table-1 [13-19] summarizes some common fish bacterial diseases [13-17], their causative agents [13-17], the main hosts [13-17], and the commercial vaccines available [18,19]. The table clearly shows that these bacteria are not host-specific, indicating that cross infections can occur between fish infected with different pathogens, and that such diseases are induced by several factors.

**Table-1:** List of fish bacterial diseases, the causative agents and main hosts, and some of the vaccine commercially available in the market.

Diseases^1^	Pathogen^1^	Main hosts^1^	Type of vaccine^2^	Trade name^2^	Vaccination route^2^
BKD	*Renibacterium salmoninarum*	Salmonids	*Arthrobacter* vaccine	Renogen	Injection
Edwardsiellosis/Redpest	*Edwardsiella tarda*	Salmon, catfish, carps, turbot, flounder, eel, tilapia	n.a	n.a	n.a
Edwardsiellosis/Enteric septicemia	*Edwardsiella ictaluri*	Channel catfish, freshwater catfish, striped catfish, brown bullhead, *Donio* spp.	*Edwardsiella ictaluri* vaccine, avirulent live culture	AquaVac-ESC™	Immersion
Flavobacteriosis/Columnaris	*Flavobacterium. columnare*, *Flavobacterium maritimus*	Cyprinids, salmonids, catfish carp, trout, perch, tilapia	*Flavobacterium columnare* bacterin	FryVacc1	Immersion
			*Flavobacterium columnare* vaccine, avirulent live culture	AquaVac-Col™	Immersion
Furunculosis	*Aeromonas salmonicida*	Salmons, trout, flounder, turbot, carp, tilapia, sole	*Aeromonas salmonicida* bacterin	Furogen Dip	Injection
			IROMP antigens of *Aeromonas salmonicida*	AquaVac^®^ FNM	Injection
			Inactivated strain of *Listonella (Vibrio) anguillarum* serovar O1, *Listonella (Vibrio) anguillarum* serovar O2, *Aeromonas salmonicida* subsp *salmonicida*, *Vibrio salmonicida*, and *Moritella viscosa* and surface protein from IPN virus serotype spp.	Norvax^®^ Minova 6	Injection
			Infectious salmon anemia virus vaccine-*Aeromonas salmonicida-Vibrio anguillarum-ordalii salmonicida* bacterin	Forte V1	Injection
Lactococcosis	*Lactococcus garvieae*	Salmonids, seabream, seabass, *Seriola* spp.	Inactivated *Lactococcus garvieae*	Amalin™ Rensa	Oral
Motile Aeromonas Septicaemia	*Aeromonas hydrophila*, *Aeromonas salmonicida*	Salmonids, bass, carp, trout, eel, sturgeon, tilapia	n.a	n.a	n.a
Pasteurellosis	*Photobacterium. damselae* spp. *piscicida*	Seabream, seabass, ayu, yellowtail, carp, sturgeon, hybrid striped bass, tuna, cobia, snakehead	Inactivated *Photobacterium damsel*	AquaVac Photobac Prime™	Immersion/Oral
			Inactivated strain *Listonella* (*Vibrio anguillarum* (biotype I and II) and *Photobacterium damselae* spp. *piscicida*	AquaVac^®^ *Vibrio Pasteurella*	Injection
Piscirickettsiosis/Rickettsial septicemia	*Piscirickettsia salmonis*	Salmonids, trout, seabass, tilapia	n.a	n.a	n.a
Streptococcosis	*Streptococcus agalactiae*, *Streptococcus iniae*, *Streptococcus dysgalactiae*, *Streptococcus parauberis*, *Streptococcus phocae*	Grouper, salmonids, turbot, flounder, sturgeon, amberjack, yellow tail, red porgy, barramundi, rabbitfish, seabass, seabream, hybrid striped bass, catfish, mullet, pomfret, tilapia, koi, carp	*Streptococcus agalactiae* biotype 2 bacterin	^1^AquaVac Garvetil/AquaVac Garvetil Oral; AquaVac^®^ Strep Sa;	Immersion/Oral
Vibriosis	*Vibrio alginolyticus*, *Vibrio parahaemolyticus*, *Vibrio vulnificus*, *Vibrio anguillarum*	Most marine fish, salmonids, groupers, cods, red seabream, gilt-head sea bream, Japanese flounder, summer flounder, amberjack, halibut, yellowtail, seabass, seriolla, milkfish, horse mackerel, cobria, sole, eel, tilapia	*Streptococcus iniae* bacterin	Norvax^®^ Strep Si	Immersion/Injection
			*Vibrio anguillarum-ordalii* bacterin	Vibrogen 2	Immersion
			Inactivated *Vibrio anguillarum* 01 and 02 (*Vibrio ordalii*)	AquaVac^®^ *Vibrio*, *AquaVac*^®^ *Vibrio* Oral Boost	Immersion/Oral
			Inactivated strain *Listonella* (*Vibrio anguillarum* (biotype I and II) and *Photobacterium damselae* spp. *piscicida*	AquaVac^®^ *Vibrio Pasteurella*	Injection
			*Aeromonas salmonicida Vibrio anguillarum ordalii salmonicida* bacterin	Lipogen Forte	Injection
			Infectious salmon anemia virus vaccine *Aeromonas salmonicida Vibrio anguillarum ordalii salmonicida* bacterin	Forte V1	Injection
Yersiniosis/Enteric redmouth	*Yersinia ruckeri*	Salmonids, trout, eel, minnows, tilapia	*Yersinia ruckeri* bacterin	Ermogen; AquaVac^®^ ERM; AquaVac^®^ ERM Oral; AquaVac^®^ RELERA™	Immersion/Oral

1 Source: [13,14,16,17],

2 [18,19]. BKD=Bacterial kidney disease, IROMP=Iron regulated outer membrane protein

## Disease Prevention in Aquaculture

A preventive approach is the best course of action to overcome disease outbreaks in aquaculture. Scientific research on health and environmental constraints of the hosts, the pathogenesis of diseases, and prevention strategies must be well addressed. To date, prevention and control of diseases rely on antibiotics and other chemicals for treatment. However, the use of antibiotics in the management of fish diseases is not recommended, due to their negative impacts on aquatic environments, such as the development of antimicrobial drug resistance in pathogenic strains [3,20]. Instead of chemical disease control strategies, biological strategies can be applied. In addition, biosecurity measures are important in preventing the occurrence of disease-causing agents in aquaculture. This includes stringent quarantine measures, egg disinfection, fish traffic control, water treatments, clean feed, and disposal of carcasses [1]. Biological control and prevention of infectious diseases in aquaculture are often achieved with the application of vaccines. However, the success rate of vaccination depends on the development of protective vaccines and their proper application [21].

## Bacterial Fish Vaccine Usage in Aquaculture

Vaccines are a powerful tool, proven to provide an easy, and cost-effective preventive solution to fish diseases [6,16,22,23]. Vaccines, in addition to reducing antibiotic dependence and the severity of losses incurred due to diseases, are known to improve fish health, reduce disease outbreaks, and provide long-lasting protection against diseases, while leaving no harmful residues in the product or the environment [6,16,22,23]. More importantly, vaccines do not have any side effects, in terms of inducing pathogen resistance, compared to antibiotics [6,22,24]. However, once a disease outbreak occurs, the application of vaccines is pointless [22].

Vaccines play a significant role in inducing an immune response and increasing the resistance to diseases in the host’s system. The immune system of the host will remain sensitized and ready to respond to the pathogens encountered by the host [22]. In fish vaccine development, studies have focused on vaccine formulation, development of vaccination regimes, and the protective efficacy of these vaccines. Several types of vaccines, such as killed whole-cell [25-27], live-attenuated [28-34], DNA vaccine [35,36], subunits [37-39], anti-idiotypic [40], and toxoid vaccines [22], have already been developed. To date, most commercially available and authorized vaccines used in the aquaculture industry are killed whole-cell vaccines. Other types of vaccines are being developed, but they are still at the experimental stage or under live animal clinical studies.

### Killed whole-cell vaccines

The killed whole-cell vaccine, also known as bacterin, is a common type of bacterial vaccine. Bacterin and inactivated vaccines are commercially available and authorized to be used in the aquaculture industry [41]. These vaccines are created using physical (heat) and chemical mutagenesis, usually with formalin or chloroform [42]. Adjuvants are often added to these vaccines, as immune potentiators or vaccine carriers [43], to increase the vaccine’s efficiency of inducing a potent immune response [27,42]. Firdaus-Nawi *et al*. [44] demonstrated the increased effectiveness of killed whole-cell vaccines added with adjuvants. They found that the addition of incomplete Freund’s adjuvant (20% v/v) to a formalin-killed *Streptococcus agalactiae* vaccine resulted in 100% survival of red tilapia intraperitoneally (IP) challenged with *S. agalactiae*, compared to only 50% survival with non-adjuvanted vaccines [44]. Huang *et al*. [27] used two types of adjuvants, the ISA763A – a non-mineral oil emulsion formulated as a metabolizable adjuvant, and the AS-F – a mineral oil-based adjuvant which is not yet commercialized, in the formalin-inactivated whole-cell vaccine of *Streptococcus iniae*; intraperitoneal infection in vaccinated *Epinephelus coioides* resulted in 100% survival [27].

### Live-attenuated vaccines

Besides killed vaccines, live-attenuated vaccines are under strong consideration to be commercialized as fish vaccines due to their advantages. Scientific studies are being increasingly focused on live-attenuated vaccines due to several reasons, such as the virulence factors displayed on the surface, ease of culturing, cheap production, and clear genetic background [45]. Furthermore, live-attenuated vaccines can induce cell-mediated and humoral antibodies, in addition to mucosal immunity [46]. Therefore, they stimulate greater adaptive immune protection in fish, compared to that induced by inactivated bacterins or subunit vaccines [47]. Live-attenuated vaccines carry native antigenic structures that are normally expressed by pathogens *in vivo*. This causes a self-limiting infection that mimics, on a smaller scale, the real infection induced following natural exposure [48]. Immune responses stimulated by such “mimic” infections closely resemble those detected in a normal infection. The antigens produced during a live infection may respond differently than those administered in the form of subunit vaccines [49].

Live-attenuated vaccines offer a prolonged and unaltered antigen presentation, which stimulates humoral and cell-mediated immune responses [47]. They are incapable of producing clinical disease; however, they can colonize appropriate sites and stimulate secretory responses [49]. Furthermore, they do not require adjuvants, and only single or few doses are needed during vaccination [50]. Attenuation was traditionally achieved through the induction of random mutation(s) by serial passage of the virulent strain in specific antibiotics [41,51,52], or on laboratory media [32]. In contrast, the modern attenuation strategy uses genetic modification techniques, such as random transposon recombination or allelic exchange replacement [47]. The latter technique has gained much interest as it offers more stable and definite attenuation, compared to the techniques typically used for killed whole-cell vaccines.

## Potential of Live-attenuated Vaccine in Aquaculture

### Live-attenuated vaccine using traditional attenuation strategy

A live-attenuated vaccine using selective double resistance to rifampin-streptomycin was developed against *V. anguillarum* strain VAN1000 [51]. It was tested on juvenile rainbow trout (*O. mykiss*), where it was shown to provide good homologous protection against *V. anguillarum*, but only slight protection against *Aeromonas salmonicida*. A live vaccine containing *Arthrobacter* spp. has also been successfully demonstrated to cross-protect against *Renibacterium salmoninarum*, a pathogen that causes bacterial kidney disease in salmonids [41,52]. It has been licensed for use on salmonids in North America and Chile [41].

Hu *et al*. [53] successfully attenuated a mutant, designated as strain T4DM, using a selection of rifampicin resistance from a virulent *V. harveyi* strain, T4D. The mutant strain T4DM was able to induce effective cross-species protection against both *V. harveyi* and *V. alginolyticus*, when used as a live immersion vaccine. Live-attenuated strains developed through repeated *in vitro* passage have also been shown to provide significant immune protection. Li *et al*. [32] developed the attenuated *S. agalactiae* YM001, through 840 continuous *in vitro* passages. Tilapia vaccinated with this strain (1.0×10^8^ CFU/fish of *S. agalactiae* YM001) exhibited 96.88% (injection), 67.22% (immersion), and 71.81% (oral) relative percentage survival (RPS), 15 days post-vaccination. Furthermore, the hosts challenged after 30 days showed an RPS of 93.61% (injection), 60.56% (immersion), and 53.16 % (oral) [32].

### Live-attenuated vaccine using genetic engineering strategy

Recent advances in molecular biology, immunology, and genetic engineering have offered exceptional technological developments in the fields of pathogenesis and recombinant DNA. Molecular biology and immunology further reveal information relating to the identification and characterization of pathogens and their pathogenicity [54]. Genetic engineering has made the construction of precise attenuated vaccines possible. Site-directed mutagenesis (SDM) is a reliable strategy to obtain a well-defined deletion, insertion, or addition in targeted genes [55]. Thus, directed attenuation can be achieved through insertion, deletion, or disruption in the metabolic pathway(s) or virulence gene(s) responsible for pathogenicity [46,56,57]. Live-attenuated vaccines developed using this new approach is remarkably potential and more efficient than bacterins in eliciting a protective immune response [31].

Ma *et al*. [28] successfully developed a two-strain polyvalent live-attenuated vaccine through genetic engineering and molecular biology, instead of the traditional serial passage technique. The strains, designated as MVAV6203 (deletion of aromatic amino acid and folic acid synthesis gene, *ΔaroC*) and MVAV6204 (deletion of aromatic amino acid and folic acid synthesis gene and siderophore anguibactin, *ΔaroCΔangE*), were developed from the *V. anguillarum* strain MVM425. The results revealed a 100% protection in *Epinephelus* spp. and *Paralichthys* spp. against *V. anguillarum* and *V. alginolyticus* infections, after being vaccinated with the two attenuated strains through intraperitoneal and immersion routes [28]. This indicated that the deletion of the target gene prevented the synthesis of an aromatic acid, folic acid, and siderophore anguibactin, thus reducing the strains’ ability to colonize in nature and also in the fish body [28].

In another study, flounders (*Paralichthys olivaceus*) vaccinated with 10^7^ CFU/ml attenuated strain *ΔaroAΔesrB* exhibited 100% RPS against 10^7^ CFU/ml *Edwardsiella tarda* [32]. This implies that live-attenuated vaccines can stimulate a cell-mediated immune response, while non-living vaccines cannot. Mou *et al*. demonstrated that isocitrate dehydrogenase mutation in *V. anguillarum* resulted in virulence attenuation and subsequent protection in rainbow trout (*O. mykiss*) [58]. Insertional mutagenesis in isocitrate dehydrogenase (*icd*) gene of *V. anguillarum* M93Sm successfully inhibited the synthesis of α-ketoglutarate in *V. anguillarum* (XM420) *icd* mutant. After 2 weeks of immersion with *icd* mutant in 1.5% salt solutions at a dose of 4×10^6^ CFU/ml, 90% survival was recorded in *O. mykiss*, compared to 30% survival of fish immersed in its parental strain. It was found the *icd* mutant showed strong attenuation in virulence, resulting in a decrease in growth yield, when comparing to the wild type, due to its inability to synthesize α-ketoglutarate, an important component for central metabolism of the pathogen [58].

Mohd-Aris *et al*. [34] successfully developed a *V. harveyi* mutant by protease deletion, as a candidate live-attenuated vaccine against vibriosis in *Epinephelus fuscoguttatus*. They employed SDM and allelic exchange replacement techniques to genetically attenuate the *V. harveyi* strain *MVh-vhs*. The *MVh-vhs* strain was shown to be safe when tested in the host, suggesting that the attenuation of virulence-associated protease *MVh-vhs* decreases the virulence properties. However, further IP vaccination of *E. fuscoguttatus* with a single dose of the attenuated strain at 10^5^ CFU/fish showed 52% RPS after being challenged with 10^8^ CFU/fish of the parental strain [34]. This suggests that the administration dosage during vaccination, may improve the protective efficacy of the *MVh-vhs* strain. Higher survival was observed in *Artemia salina* larvae incubated with 10^7^ CFU/mL of the live-attenuated strain *MVh-vhs*, 6 h post-incubation. Furthermore, *A. salina* larvae incubated with *MVh-vhs* (10^9^ CFU/mL) showed a higher survival rate when challenged with pathogenic *V. harveyi* (*Vh1*), *V. alginolyticus* (*VA2*), and *V. parahaemolyticus* (*FORC*_008), 24 h after incubation [59].

### Combined live-attenuated vaccines

A combined live-attenuated vaccine utilizes a “ghost” or vector to harbor foreign materials obtained from the pathogen, to express and evoke the host’s immune system [60]. The primary advantage of live-attenuated vectors is their ability to deliver multiple antigens, of different species, in a single dose. Other advantages include the mimicry of natural infection, intrinsic adjuvant characteristics, and the possibility of being administered through the mucosal route, rather than the more laborious intraperitoneal route [56]. In addition, combined vaccines can achieve high expression of antigens, due to the plasmid-mediated expression system. Table-2 [31,61-64] summarizes recent studies related to combined live-attenuation [61-64]. Goa *et al*. [31] described the capability of a combined vaccine, consisting of live-attenuated *E. tarda* WED and *V. anguillarum* MVAV6203, to evoke better immune-mediated protection in turbot (*Scophthalmus maximus*) and zebrafish (*Danio rerio*) against *E. tarda* EIB202 and *V. anguillarum* MVM425, with the activation of toll-like receptors and Class I and Class II major histocompatibility complexes.

**Table-2:** Combined live-attenuated vaccine of *Vibrio* spp.

Bacterial vector	Method of combined vaccine	Protection against	Research findings	References
Avirulent *Vibrio anguillarum* (MVAV6203)	Inoculation of *Pseudomonas syringae* (ICMP3023) *inaV* gene	*Vibrio anguillarum* and *Pseudomonas syringae*	The expression of foreign antigen in vector was expressed both in cytoplasms an OMP of vector	[61]
Avirulent *Vibrio anguillarum* (MVAV6203)	Inoculation of *Edwardsiella tarda* pUTatgap plasmid	*Vibrio anguillarum* and *Edwardsiella tarda*	Survival of 80% and 67% when challenged with *Vibrio anguillarum* and *Edwardsiella tarda*, respectively, in turbot (*Scophthalmus maximus*)	[62]
Avirulent *Vibrio anguillarum* (MVAV6203) and *Edwardsiella tarda* (WED)	Polyvalent live attenuated vaccine	*Vibrio anguillarum* and *Edwardsiella tarda*	Survival of 90% and 70% when challenged with *Vibrio anguillarum* and *Edwardsiella tarda*, respectively, in zebrafish (*Danio rerio*)	[31]
Avirulent *Vibrio anguillarum* (MVAV6203)	Inoculation of *Aeromonas hydrophila* (LSA34) GAPDH strain AV/pN-gapA	*Vibrio anguillarum* and *Aeromonas hydrophila*	Survival of 87% and 67% when challenged with *Vibrio anguillarum* and *Aeromonas hydrophila*, respectively, in turbot (*Scophthalmus maximus*)	[63]
Avirulent *Vibrio anguillarum* (MVAV6203)	Inoculation of *Edwardsiella tarda* (EIB202) EseB OMP	*Vibrio anguillarum* and *Edwardsiella tarda*	Survival of 100% and 0% when challenged with *Vibrio anguillarum* and *Edwardsiella tarda*, respectively, in zebrafish (*Danio rerio*)	[64]

## Constraints that Limit the Potential of Live Vaccines

### Risks in the protective efficacy of live vaccines

Despite the remarkable advantages of live vaccines, a few disadvantages have been discerned. Although attenuation strategies produce attenuated isolates, the isolates only persist for a short duration, between 24 and 72 h, and fail to stimulate adequate immunity in young fish [46]. Live vaccines also have the risk of producing low-grade infections when the vaccine agents replicate in the hosts. They may even result in systemic symptoms, displaying some features of the original infection [65].

### Stability and maintenance of live vaccines

Compared to killed vaccines, live vaccines are less stable and have a shorter shelf life. This may be due to the nature of live cells, which are easily affected by environmental factors, for example, susceptibility to damages or destructions by high temperatures due to heat-labile characteristics [50]. It is important to provide a cold or refrigerated environment (2-8°C) during handling, storage, and distribution of live vaccines, to ensure stability throughout their designated shelf life [66]. This leads to higher operational and handling costs, thus adding to the total expenses for vaccination.

### Commercialization process and legislation hurdles

In addition to operational and handling costs, extensive research, such as risk assessments and clinical testing of live-attenuated bacteria, requires huge investments before vaccine registration [67]. All costs incurred during the development of live vaccines greatly influence their market price [50]. As a result, most live vaccines are still at the research stage. Another issue with live vaccines is the regulatory hurdles in vaccine registration [57]. The procedure from research to obtaining a valid license for this type of vaccine is rather long and often exorbitant. Moreover, legislation on the control and administration of vaccines varies greatly from country to country [68]. For example, in the EU, the USA, and Japan, a licensed vaccine is required to be included while importing aquaculture products [69]. Therefore, tedious regulatory hurdles cannot be neglected. Concerns related to costing, budgeting, stable formulation, fill-finish step, and economical production are some of the limitations in the application of live vaccines, especially if the vaccine is targeted for use by aquaculture practitioners or farmers in developed countries [66,70].

### Stability in virulence attenuation properties

Another limitation of using a live vaccine is the possibility of back-mutation and reversion to its virulent phenotype [8,67,68] which may occur due to changes in the bacteria, or compromising conditions in the host. The attenuated strains might be well tolerated by healthy individuals, but some may induce auto-immune responses, causing local inflammation and other adverse reactions [71]. Thus, a strategy to reduce virulence reversion during live vaccine development is to attenuate multiple genes instead of a single gene [57]. Furthermore, there is a risk of introducing pathogenic strains from live-attenuated agents into the aquatic environment, which might become a pathogenic source for other species [72]. Immersion vaccination has been preferred by most fish farmers to date [73], as the processing and vaccine application is easier. However, developments in vaccine production and processing technologies, storage, and delivery methods are required.

### Negative public perception

All vaccines, including live vaccines, carry some risk, even when they present an excellent track record in terms of safety in human and veterinary use [57]. The long-term challenge for live vaccines is to infuse understanding and shape public perception [65]. Live vaccines are often the subject of unsubstantiated accusations by anti-vaccine movements; they are faced with public resistance and voiced against strongly as they are genetically modified [57,65]. Therefore, it is necessary to properly design, in addition to conducting efficacy and other related tests to gather essential data to refute false claims raised by the public. Safety aspects must be prioritized to diminish the undesirable impacts of live vaccines. This approach will greatly help to rectify and fortify public trust toward vaccination, which is important for the aquaculture sector, to exploit the benefits of live vaccines [65].

### Safety issues and environmental release

There is also a risk for this type of vaccine to spread from a vaccinated to an unvaccinated individual, due to the release of the pathogen in the environment, or exposure to non-target animals [50,57]. For example, a worst-case scenario would be where water samples treated with attenuated strains are accidentally released into the open environment during disposal. Under these circumstances, safety of the environment and the residing population is jeopardized, as the attenuated strain can cause infections in the human population [46]. The potential risk of admission and transmission needs to be scrutinized, especially by the person in charge of the vaccination process. Thus, the evaluation of the potential impact of environmental release and the risk of horizontal gene transfer is highly critical [50]. It is crucial to monitor the biosafety aspects of the attenuated vaccines applied in aquaculture. As proposed by Ma *et al*. [28], the genetic background of the mutation must be clear, a double deletion should be considered to eliminate the reversion of virulence characteristics, and the attenuation should be definite, so that the environmental safety and controllability of the vaccine are feasible, and the possibility of exposing the pathogen to the environment can be minimized.

## Conclusion and Future Prospects

Vaccination strategy is an integral part of comprehensive fish health management. It is the best preventive strategy to combat the spread of fish diseases by inducing defense mechanisms against the risk of bacterial disease outbreaks. Hence, fundamental knowledge of diseases and pathogen profiles, in addition to the basic economic background of operational costs, is an essential requirement in the design of suitable vaccination strategies. There are promising indicators that live vaccines have great potential to be further exploited as alternative vaccines. However, each presumable benefit and implication must be carefully assessed when designing a new candidate live vaccine. In spite of the potential problems and undesired ramifications, the holistic advantages still outweigh the disadvantages, thus, endeavoring to develop new live vaccines is a worthy investment. It is strongly suggested that all possible limitations must be critically addressed before employing live-attenuated vaccines in aquaculture sectors. Overall market demand, integration of suitable vaccination regimes, and good disease management unequivocally facilitate improvement in fish survival rates, further boosting the production of the aquaculture industry.

## Authors’ Contributions

AM conceived and framed the main idea of this manuscript. AM and MHNM prepared the first draft. The first draft was read, criticized, and corrected by MYI, MZ, and HMD. AM proofread the second draft and finalized the manuscript. All authors have read and approved the final manuscript.

## References

[1] Assefa A, Abunna F (2018). Review article:Maintenance of fish health in aquaculture:A review of epidemiological approaches for prevention and control of infectious disease of fish. Vet. Med. Int.

[2] FAO (Food and Agriculture Organization of the United Nations) (2016). The State of World Fisheries and Aquaculture:Contributing to Food Security and Nutrition for All.

[3] Harikrishnan R, Balasundaram C, Heo M.S (2011). Fish health aspects in grouper aquaculture. Aquaculture.

[4] Bowater R.O, Forbes-Faulkner J, Anderson I.G, Condon K, Robinson B, Kong F, Gilbert G.L, Reynolds A, Hyland S, McPherson G, Brien J.O, Blyde D (2012). Natural outbreak of *Streptococcus agalactiae* (GBS) infection in wild giant Queensland grouper *Epinephelus lanceolatus* (Bloch), and other wild fish in northern Queensland, Australia. J. Fish Dis.

[5] Albert V, Ransangan J (2013). Effect of water temperature on susceptibility of cultured marine fish species to vibriosis. Int. J. Res. Pure Appl. Microbiol.

[6] Bondad-Reantaso M.G, Subasinghe R.P, Arthur J.R, Ogawa K, Chinabut S, Adlard R, Tan Z, Shariff M (2005). Disease and health management in Asian aquaculture. Vet. Parasitol.

[7] FAO (Food and Agriculture Organization of the United Nations) (2017). Fishery and Aquaculture Statistics:Global Production by Production Source, 1950-2015 (FishstatJ).

[8] Adams A, Aoki T, Berthe F.C.J, Grisez L, Karunasagar I, Bondad-Reantaso M.G, Mohan C.V, Margaret C, Subasinghe R.P (2008). Recent technological advancements on aquatic animal health and their contributions toward reducing disease risks a review. Diseases in Asian aquaculture VI. Fish Health Section.

[9] Dey M.M, Briones R.M, Garcia Y.T, Nissapa A, Rodriguez U.P, Talukder R.K, Senaratne A, Omar I.H, Koeshendrajana S, Khiem N.T, Yew T.S, Weimin M, Jayakody D.S, Kumar P, Bhatta R, Haque M.S, Rab M.A, Chen O.L, Luping L, Paraguas F.J (2008). Strategies and Options for Increasing and Sustaining Fisheries and Aquaculture Production to Benefit Poorer Households in Asia. WorldFish Center Studies and Reviews No. 1823.

[10] Somga J.R, Somga S.S, Reantaso M.B, Arthur J.R, Phillips M.J, Subasinghe R.P, Reantaso M.B, MacRae I.H (2002). Impacts of disease on small-scale grouper culture in the Philippines. Primary Aquatic Animal Health Care in Rural, Small-Scale, Aquaculture Development.

[11] NASS-USDA (2019). Trout Production.

[12] Shefat S.H.T (2018). Vaccines for infectious bacterial and viral diseases of fish:A review. J. Bacteriol. Infect. Dis.

[13] Pridgeon J.W, Klesius P.H (2012). Review:Major bacterial diseases in aquaculture and their vaccine development. CAB Int. Rev.

[14] Austin B, Austin D.A (2007). Bacterial Fish Pathogens:Diseases of Farmed and Wild Fish.

[15] Liu L, Ge M, Zheng X, Tao Z, Zhou S, Wang G (2016). Investigation of *Vibrio alginolyticus*
*V. harveyi* and *V. parahaemolyticus* in large yellow croaker *Pseudosciaena crocea* (Richardson) reared in Xiangshan Bay, China. Aqua. Rep.

[16] Grisez L, Tan Z, Walker P, Lester R, Bondad-Reantaso M.G (2005). Vaccine development for Asian aquaculture. Diseases in Asian aquaculture V. Proceedings of the Fifth Symposium in Asian Aquaculture Held on 25-28 November 2005.

[17] Singh S, Bala M, Kanwar S.S (2014). Bacterial and viral diseases in fish &shrimps. Int. J. Microbiol. Allied Sci.

[18] Bowker J.D, Trushenski J.T (2016). Guide to Using Drugs, Biologics, and Other Chemicals in Aquaculture. American Fisheries Society Fish Culture Section.

[19] http://www.aqua.merck-animal-health.com/products/vaccines.aspx.

[20] Xiong W, Sun Y, Zhang T, Ding X, Li Y, Wang M, Zeng Z (2015). Antibiotics, antibiotic resistance genes, and bacterial community composition in freshwater aquaculture environment in China. Microb. Ecol.

[21] Lillehaug A, Lillehaug A.G.R, Evensen Ø (2014). Vaccination Strategies and Procedures in Fish Vaccination.

[22] Sudhagar A, Khan F, Prabu L (2016). Fish Vaccination:A Health Management Tool for Aquaculture.

[23] Ina-Salwany M.Y, Al-Saari N, Mohamad A, Mursidi F.A, Mohd-Aris A, Amal M.N.A, Kasai H, Mino S, Sawabe T, Zamri-Saad M (2018). Vibriosis in fish:A review on disease development and prevention. J. Aquat. Anim. Health.

[24] Toranzo A, Romalde J, Magariños B, Barja J, Rogers C, Basurco B (2009). Present and future of aquaculture vaccines against fish bacterial diseases. The Use of Veterinary Drugs and Vaccines in Mediterranean Aquaculture. Zaragoza:CIHEAM. Vol. 88. Options Méditerranéennes:Série A.

[25] Karsi A, Lawrence M.L, Abdelhamed H (2017). Live Attenuated *Edwardsiella ictaluri* Vaccine and Method of Using the Same. U.S. Patent Application US2017/0065695 A1.

[26] Nguyen H.T, Nguyen T.T.T, Tsai M, Ya-Zhen E (2017). A formalin-inactivated vaccine provides good protection against *Vibrio harveyi* infection in Orange-spotted grouper (*Epinephelus coioides*). Fish Shellfish Immun.

[27] Huang H.Y, Chen Y.C, Wang P.C, Tsai M.A, Yeh S.C, Liang H.J, Chen S.C (2014). Efficacy of a formalin-inactivated vaccine against *Streptococcus iniae* infection in the farmed grouper *Epinephelus coioides* by intraperitoneal immunization. Vaccine.

[28] Ma Y, Zhang Y, Zhao D (2008). Polyvalent Attenuated Live Vaccine for Preventing and Curing Vibriosis of Cultivated Fish. US Patent 0274136 A1.

[29] Hu Y, Sun L (2011). A bivalent *Vibrio harveyi* DNA vaccine induces strong protection in Japanese flounder (*Paralichthys olivaceus*). Vaccine.

[30] Pridgeon J.W, Klesius P.H, Yildirim-Aksoy M (2013). Attempt to develop live attenuated bacterial vaccines by selecting resistance to gossypol, proflavine hemisulfate, novobiocin or ciprofloxacin. Vaccine.

[31] Gao Y, Wu H, Wang Q, Qu J, Liu Q, Xiao J, Zhang Y (2014). A live attenuated combination vaccine evokes effective immune-mediated protection against *Edwardsiella tarda* and *Vibrio anguillarum*. Vaccine.

[32] Li J, Mo Z, Li G, Xiao P, Huang J (2015). Generation and evaluation of virulence attenuated mutants of *Edwardsiella tarda* as vaccine candidates to combat edwardsiellosis in flounder (*Paralichthys olivaceus*). Fish Shellfish Immunol.

[33] Wang R, Li L, Huang Y, Luo F, Liang W, Gan X, Huang T, Lei A, Chen M, Chen L (2015). Comparative genome analysis identifies two large deletions in the genome of highly-passaged attenuated *Streptococcus agalactiae* strain YM001 compared to the parental pathogenic strain HN016. BMC Genomics.

[34] Mohd-Aris A, Zamri-Saad M, Daud H.M, Yusof M.T, Ina-Salwany M.Y (2019). *Vibrio harveyi* protease deletion mutant as a live attenuated vaccine candidate against vibriosis and transcriptome profiling following vaccination for *Epinephelus fuscoguttatus*. Aquacult. Int.

[35] Sepúlveda D, Lorenzen N (2016). Can VHS virus bypass the protective immunity induced by DNA vaccination in rainbow trout?. PLoS One.

[36] Rauta P.R, Nayak B, Monteiro G.A, Mateus M (2017). Design and characterization of plasmids encoding antigenic peptides of *Aha1* from *Aeromonas hydrophila* as prospective fish vaccines. J. Biotechnol.

[37] Zhang W.W, Sun K, Cheng S, Sun L (2008). Characterization of *DegQVh* a serine protease and a protective immunogen from a pathogenic *Vibrio harveyi* strain. Appl. Environ. Microbiol.

[38] Hamod A.M, Nithin M.S, Shukur N.Y, Karunasagar I, Karunasagar I (2012). Outer membrane protein K as a subunit vaccine against *Vibrio anguillarum*. Aquacultures.

[39] Nehlah R, Ina-Salwany M.Y, Zulperi Z (2016). Antigenicity analysis and molecular characterization of two outer membrane proteins of *Vibrio alginolyticus* strain VA2 as vaccine candidates in Tiger Grouper culture. J. Biol. Sci.

[40] Qin Y.X, Yan Q.P (2010). The Antigenicity of the flagellin, Outer Membrane Proteins and Lipopolysaccharide of *Vibrio harveyi*. In 2010 International Conference on Bioinformatics and Biomedical Technology (ICBBT).

[41] Sommerset I, Krossøy B, Biering E, Frost P (2005). Vaccines for fish in aquaculture. Expert Rev. Vaccines.

[42] Tafalla C, Bogwald J, Dalmo R.A (2013). Adjuvants and immunostimulants in fish vaccines:Current knowledge and future perspectives. Fish Shellfish Immunol.

[43] Anderson D.P, Jeney G (1992). Immunostimulants added to injected *Aeromonas salmonicida* bacterin enhance the defense mechanisms and protection in rainbow trout (*Oncorhynchus mykiss*). Vet. Immunol. Immunopathol.

[44] Firdaus-Nawi M, Yusoff S.M, Yusof H, Abdullah S.Z, Zamri-Saad M (2013). Efficacy of feed-based adjuvant vaccine against *Streptococcus agalactiae* in *Oreochromis* spp. in Malaysia. Aquac. Res.

[45] Ye N, Wu H, Zhang Y (2016). Maternal transfer and protection role in zebrafish (*Danio rerio*) offspring following vaccination of the brood stock with a live attenuated *Vibrio anguillarum* vaccine. Aquac. Res.

[46] Shoemaker C.A, Klesius P. H (2009). Use of modified live vaccines in aquaculture. J. World Aquac. Soc.

[47] Wang J, Zou L.L, Li A.X (2014). Construction of a *Streptococcus iniae* sortase A mutant and evaluation of its potential as an attenuated modified live vaccine in Nile tilapia (*Oreochromis niloticus*). Fish Shellfish Immunol.

[48] Pore D, Chowdhury P, Mahata N, Pal A, Yamasaki S, Mahalanabis D, Chakrabarti M.K (2009). Purification and characterization of an immunogenic outer membrane protein of *Shigella flexneri*2a. Vaccine.

[49] Brown F, Dougan G, Hoey E.M, Martin S.J, Rima B.K, Trudgett A (1993). Vaccine Design. Molecular Medical Science Series.

[50] Ebensen T, Link C, Guzmán C.A, Kaufmann S.H.E (2004). Classical and novel vaccination strategies:A comparison. Novel Vaccination Strategies.

[51] Norqvist A, Hagstrom A, Wolf-Watz H (1989). Protection of rainbow trout against vibriosis and furunculosis by the use of attenuated strains of *Vibrio anguillarum*. Appl. Environ. Microbiol.

[52] Håstein T, Gudding R, Evensen Ø (2005). *Bacterial vaccines for fish*- An update of the current situation worldwide. Midtlyng PJ, editor:Progress in Fish Vaccinology. Dev. Biol.

[53] Hu Y.H, Deng T, Sun B.G, Sun L (2012). Development and efficacy of an attenuated *Vibrio harveyi* vaccine candidate with cross protectivity against *Vibrio alginolyticus*. Fish Shellfish Immunol.

[54] Li N, Yang Z, Bai J, Fu X, Liu L, Shi C, Wu S (2010). A shared antigen among *Vibrio* species:Outer membrane protein-OmpK as a versatile vibriosis vaccine candidate in orange-spotted grouper (*Epinephelus coioides*). Fish Shellfish Immunol.

[55] IDT (Integrated DNA Technology) (2011). Mutagenesis Application Guide:Experimental Overview, Protocol, Troubleshooting. Integrated DNA Technologies.

[56] Xiao J, Chen T, Wang Q, Liu Q, Wang X, Lv Y, Wu H, Zhang Y (2011). Search for live attenuated vaccine candidate against Edwardsiellosis by mutating virulence-related genes of fish pathogen *Edwardsiella tarda*. Lett. Appl. Microbiol.

[57] Loessner H, Schwantes A, Hamdorf M, Komor U, Leschner S, Weiss S, von Gabain A, Klade C (2012). Employing live microbes for vaccine delivery. Development of Novel Vaccine.

[58] Mou X, Spinard E.J, Hillman S.L, Nelson D.R (2017). Isocitrate dehydrogenase mutation in *Vibrio anguillarum* results in virulence attenuation and immunoprotection in rainbow trout (*Oncorhynchus mykiss*). BMC Microbiol.

[59] Chin Y.K, Al-Saari N, Mohd-Aris A, Zamri-Saad M, Ina-Salwany M.Y (2019). Administration of live-attenuated vaccine of *Vibrio harveyi* to improve survival of gnotobiotic brine shrimp (*Artemia salina*) model against multiple *Vibrio* infection. Int. J. Biosci.

[60] Choi S.H, Nam Y.K, Kim K.H (2010). Novel expression system for combined vaccine production in *Edwardsiella tarda* ghost and cadaver cells. Mol. Biotechnol.

[61] Xu Y, Liu Q, Zhou L, Yang Z, Zhang Y (2008). Surface display of GFP by *Pseudomonas syringae* truncated ice nucleation protein in attenuated *Vibrio anguillarum* strain. Mar. Biotechnol.

[62] Xiao Y, Liu Q, Chen H, Zhang Y (2011). A stable plasmid system for heterologous antigen expression in attenuated *Vibrio anguillarum*. Vaccine.

[63] Zhao Y, Liu Q, Wang X, Zhou L, Wang Q, Zhang Y (2011). Surface Display of *Aeromonas hydrophila* GAPDH in Attenuated *Vibrio anguillarum* to develop a novel multivalent vector vaccine. Mar. Biotechnol.

[64] Wang Q, Yang Z, Liu Q, Zhang Y (2009). Surface-exposed expression of *Edwardsiella tarda* EseB in live attenuated *Vibrio anguillarum* based on novel surface display systems. Aquac. Res.

[65] Goldman M, Lambert P-L, Kaufmann S. H. E (2004). Immunological safety of vaccines:Facts, hyphotheises and allegations. Novel Vaccination Strategies.

[66] Schlegl R, Hahn R, von Gabain A, Klade C (2012). Purification and formulation:Silent but important players in vaccine development. Development of Novel Vaccine.

[67] Frey J (2007). Biological safety concepts of genetically modified live bacterial vaccines. Vaccine.

[68] Ellingsin K, Gudding R (2011). The Potential to Increase use of the 3Rs in the Development and Validation of Fish Vaccines. Report from the National Veterinary Institute, Oslo.

[69] Meeusen E.N.T, Walker J, Peters A, Pastoret P.P, Jungersen G (2007). Current status of veterinary vaccines. Clin. Microbiol. Rev.

[70] Thorarisson R, Powell D (2006). Effects of disease risk, vaccine efficacy and market price on the economics of fish vaccination. Aquaculture.

[71] Jorge S, Dellagostin O.A (2017). The development of veterinary vaccines:A review of traditional methods and modern biotechnology approaches. Biotechnol. Res. Innovation.

[72] Munangandu H.M, Mutoloki S, Evensen Ø (2015). An overview of challenges limiting the design of protective mucosal vaccines for finfish. Front. Immunol.

[73] Sudheesh P.S, Cain K.D (2017). Prospects and challenges of developing and commercializing immersion vaccines for aquaculture. Int. Biol. Rev.

